# Theranostic Imaging Surrogates for Targeted Alpha Therapy: Progress in Production, Purification, and Applications

**DOI:** 10.3390/ph16111622

**Published:** 2023-11-17

**Authors:** Bryce J. B. Nelson, John Wilson, Jan D. Andersson, Frank Wuest

**Affiliations:** 1 Department of Oncology, University of Alberta, 11560 University Ave., Edmonton, AB T6G 1Z2, Canada; bjnelson@ualberta.ca (B.J.B.N.); john.wilson2@albertahealthservices.ca (J.W.); jan.andersson@albertahealthservices.ca (J.D.A.); 2 Edmonton Radiopharmaceutical Center, Alberta Health Services, 11560 University Ave., Edmonton, AB T6G 1Z2, Canada; 3 Cancer Research Institute of Northern Alberta, University of Alberta, Edmonton, AB T6G 2E1, Canada

**Keywords:** targeted alpha therapy, alpha particle therapy, PET imaging, SPECT imaging, targeted radionuclide therapy, theranostics, actinium-225, lanthanum-133, lead-212, lead-203

## Abstract

This article highlights recent developments of SPECT and PET diagnostic imaging surrogates for targeted alpha particle therapy (TAT) radiopharmaceuticals. It outlines the rationale for using imaging surrogates to improve diagnostic-scan accuracy and facilitate research, and the properties an imaging-surrogate candidate should possess. It evaluates the strengths and limitations of each potential imaging surrogate. Thirteen surrogates for TAT are explored: ^133^La, ^132^La, ^134^Ce/^134^La, and ^226^Ac for ^225^Ac TAT; ^203^Pb for ^212^Pb TAT; ^131^Ba for ^223^Ra and ^224^Ra TAT; ^123^I, ^124^I, ^131^I and ^209^At for ^211^At TAT; ^134^Ce/^134^La for ^227^Th TAT; and ^155^Tb and ^152^Tb for ^149^Tb TAT.

## 1. Introduction

Targeted alpha therapy (TAT) involves utilizing radiopharmaceuticals to precisely eliminate malignancies with alpha particle emissions, while sparing surrounding healthy tissues. These radiopharmaceuticals consist of alpha (α)-emitting radionuclides conjugated to a biological-targeting vector such as monoclonal antibodies, peptides, and nanocarriers [1]. Key advantages of TAT include highly selective radiation delivery to the target, reduced patient side effects, and the ability to assess radiopharmaceutical uptake and, therefore, patient eligibility using a diagnostic radionuclide before therapy [2].

While beta minus (β^−^) radiopharmaceuticals employing radionuclides such as ^177^Lu have made significant advances in clinical care of advanced prostate and neuroendocrine tumors [3,4], alpha particle emissions are significantly more precise and cytotoxic than β^−^ emissions. This is attributed to the much larger size of alpha particles (7300 times the mass of electrons), their 2+ charge resulting in a highly ionized emission path, and high linear energy transfer that deposits their energy over a path length of only several cell diameters. These properties make alpha emitters ideal for combatting metastatic cancers and other systemic malignancies where traditional treatment avenues have failed [2,5,6,7].

Approximately 400 alpha-emitting radionuclides (5–100% emission intensity) are known; however, only radionuclides that possess a sufficiently long half-life, absence of long-lived toxic progeny, and feasible high-yield production routes are suitable for TAT consideration [8,9]. Radionuclides that have shown potential for TAT include ^227^Th, ^225^Ac, ^224^Ra, ^223^Ra, ^212^Pb, ^211^At, and ^149^Tb [1,2,10,11,12,13,14,15,16,17,18,19,20].

While the potency of TAT offers significantly enhanced therapeutic efficacy, TAT must be treated as a double-edged sword with the possibility of severe off-target toxicity to nontarget organs and tissues. This mandates a comprehensive understanding of the stability, pharmacokinetics, and dosimetry of any TAT radiopharmaceutical. During preclinical development, these data can be acquired from biodistribution studies in mice, where mice are sacrificed at multiple time points, and gamma-ray co-emissions are counted in the dissected organs and tissues.

Additionally, positron emission tomography (PET) and single-photon emission computed tomography (SPECT) scans can be acquired by exchanging the alpha-emitting radionuclide with a positron or gamma-ray-emitting diagnostic imaging radionuclide. This imaging–therapeutic duality is termed “theranostics”, and these PET and SPECT scans provide crucial information on dosimetry and monitor response to TAT.

Most TAT radionuclides lack or possess insufficient co-emitted positrons or gamma rays for acquiring higher-quality PET or SPECT scans. This motivated the development of chemically similar diagnostic imaging surrogates for TAT radionuclides. As the current supply of alpha-emitting radionuclides is scarce, utilizing imaging surrogates also has the potential to open more opportunities for TAT research to facilities without access to alpha-emitting radionuclides and serve as a bridge for centers planning to introduce TAT radiopharmaceuticals. Since many of these surrogates can be synthesized in existing cyclotron facilities, this can facilitate radiopharmaceutical developments. Additionally, imaging surrogates fit well into the existing research and clinical setup. As such, TAT imaging surrogates have the potential to assist the deployment of TAT radiopharmaceuticals in the clinic and accelerate the development of new TAT targeting vectors.

## 2. Properties of Ideal Imaging Surrogates for Alpha Emitters

Multiple factors determine what makes a suitable imaging surrogate for targeted alpha therapy. These include chemical properties, half-life, radioactive emission type and intensity, associated dosimetry, production ease and scalability, radionuclidic purity, economics, and radionuclide progeny considerations.

PET and SPECT scans that evaluate the pharmacokinetics and dosimetry of TAT radiopharmaceuticals are often performed with ^68^Ga and ^18^F. However, ^68^Ga, ^18^F, and other common imaging radionuclides often have substantially different chemical properties than alpha-emitting radionuclides. For some targeting vectors, this can result in differing biodistributions between the TAT radiopharmaceutical and its diagnostic counterpart [21,22,23]. Potential inconsistencies observed in diagnostic imaging scans and subsequent biodistribution of the therapeutic radiopharmaceutical could result in sub-optimal tumor dosing or unintended and destructive alpha-irradiation of healthy tissues.

Imaging surrogates should, therefore, possess a similar chemistry and half-life to ensure their biodistribution and dosimetry are similar to their paired alpha emitters. These surrogates are ideally isotopes of the same element possessing identical chemistries, such as ^226^Ac paired with ^225^Ac TAT, ^203^Pb paired with ^212^Pb TAT, ^209^At paired with ^211^At, and ^155^Tb or ^152^Tb paired with ^149^Tb TAT.

However, if suitable isotopes of the same element are not available, chemically similar elements in the same chemical group can be employed. These include ^133^La, ^132^La, or ^134^Ce/^134^La paired with ^225^Ac, ^134^Ce/^134^La, paired with ^227^Th, and ^123^I, ^124^I, or ^131^I, paired with ^211^At.

It is also preferable that the physical half-life of the imaging surrogate is similar to its TAT counterpart. This permits the acquisition of biodistribution data for the full in vivo residence of the TAT radiopharmaceutical to assist preclinical development and initial clinical validation. For TAT employing radionuclides with long physical half-lives (^225^Ac, ^223^Ra, ^224^Ra, ^227^Th) and targeting vectors with long biological half-lives, using a long-lived imaging surrogate is crucial to confirm that the radiopharmaceutical remains at the target site for the extended duration without redistributing to and irradiating healthy tissues. While additional patient radiation dose might result from using a diagnostic radionuclide with a longer half-life, some targeting vectors such as antibodies may require longer circulation times to acquire sufficient quality images. For TAT employing long-lived radionuclides and targeting vectors with short biological half-lives, a radionuclide imaging surrogate with a shorter physical half-life may be used in certain situations. This can be a valuable tool for evaluating patient dosimetry, provided that the targeting vector exhibits rapid in vivo clearance, minimal off-target binding, and the radionuclide is stably incorporated within the radiopharmaceutical. Radiopharmaceutical pretargeting approaches may reduce the advantage of selecting diagnostic and therapeutic TAT radionuclides with similar half-lives; however, it is uncertain whether most theranostic targeting vectors will employ a pretargeting approach.

Regarding radioactive emissions, it is preferable that PET imaging surrogates possess a high positron branching ratio and low positron emission energy to facilitate high-resolution PET imaging and minimal co-emitted electrons and gamma/X-rays to reduce the radioactive dose. Radionuclides with lower positron branching ratios may require additional injected activity to resolve the same quality image. For SPECT imaging, radionuclides should possess lower energy gamma rays within the optimal energy window of scanners and minimal co-emitted electrons and gamma/X-rays.

To produce imaging surrogates, sufficient cyclotron or nuclear reactor facilities are required to synthesize the radionuclide. Target material (natural or isotopically enriched) should be available in adequate quantity and enrichment to support routine production, and a favorable nuclear cross-section must exist within the capabilities of production facilities. Radionuclide production should be performed safely, create few long-lived radionuclidic impurities, and be scalable to sufficient activities that allow distribution to clinical sites. Robust chemical-purification techniques must separate the imaging surrogate from potentially hazardous target material post-irradiation. Finally, the radionuclide progeny of the imaging surrogate should be considered since this can influence imaging quality and impact radioactive waste management.

Most radionuclides used in TAT are part of decay chains where each decay results in the recoil of the daughter nucleus with energy sufficient to liberate the daughter nucleus from the chelator into solution. Additionally, the alpha particle itself may induce radiolytic damage to the radiopharmaceutical, reducing the in vivo targeting and leading to further accumulation of radioactivity in nontarget tissue. These inherent physical properties are not easily covered by the surrogates in question, so they should be considered in experimental methods and conclusions.

In this article, a selection of 13 diagnostic imaging surrogates for promising alpha-emitting radionuclides have been highlighted for their production, purification, applications, and overall strengths and limitations.

## 3. Theranostic Imaging Surrogates Proposed for Actinium-225

Actinium-225 (t_1/2_ = 9.9 d) has been explored extensively for TAT. Its long half-life permits extended dose delivery and decay via a cascade of six short-lived radionuclide progeny with four alpha particle emissions to near-stable ^209^Bi, making ^225^Ac particularly attractive for TAT. ^225^Ac studies have demonstrated efficacy in metastatic prostate cancer and neuroendocrine tumors, and additional radiopharmaceuticals are under development for other cancers [11,24,25,26,27,28,29,30] There are considerable efforts underway to significantly increase the ^225^Ac supply to meet the significant anticipated clinical demand [31,32,33,34].

However, ^225^Ac does not emit gamma rays of sufficient intensity for imaging. Although its ^213^Bi and ^221^Fr progeny possess gamma rays of suitable energy and intensity for SPECT imaging [9], the ^225^Ac activities injected into patients (~50–200 kBq/kg [11]) would be insufficient to resolve a high-quality image within a reasonable scan duration. Additionally, the supply of high-purity ^225^Ac from ^225^Ra/^225^Ac generators is limited, constraining AT development efforts [31]. While other sources of ^225^Ac from high-energy spallation reactions are available [32,35,36], these often contain a small activity of co-produced and inseparable ^227^Ac (t_1/2_ = 21 y), which complicates radioactive waste management. Therefore, the desire to enable ^225^Ac imaging and enhance research throughput motivates the development of imaging surrogates.

For SPECT imaging, ^226^Ac is an elementally matched surrogate for ^225^Ac. Radiolanthanum isotopes ^133^La, ^132^La, and ^134^La are particularly attractive for PET imaging of ^225^Ac due to the similar ionic radii of La^3+^ and Ac^3+^ (~1.03 and ~1.12 Å, respectively [37,38]) and their resulting similar chemistries. Both lanthanum and actinium possess similar chelation chemistry with chelators such as DOTA, macropa, and crown ethers, and exhibit similar in vivo biodistributions [39,40,41,42,43,44]. The subsequent sections will outline the properties, strengths, and limitations of ^133^La, ^132^La, ^134^Ce/^134^La, and ^226^Ac.

### 3.1. Lanthanum-133 (PET)

Lanthanum-133 (t_1/2_ = 3.9 h) has been synthesized via the ^135^Ba(p,3n)^133^La and ^135^Ba(p,2n)^133^La nuclear reactions on medical cyclotrons [45]. Natural Ba metal can be used as a target material, with one study producing 231 MBq ^133^La and 166 MBq ^135^La for 500 µA·min cyclotron irradiations at 22 MeV. Subsequent chemical processing using a diglycolamide (DGA) resin produced a highly pure [^133^La]LaCl_3_ product that, when used to radiolabel DOTA and macropa chelators, achieved molar activities sufficient for preclinical and clinical application [40]. Co-production of ^135^La (t_1/2_ = 18.9 h (44)) is unavoidable using natural barium target material. While ^135^La has potential applications for Auger-Meitner electron therapy, it would add additional patient radioactive dose and is undesirable for ^133^La PET imaging applications.

Alternatively, natural or isotopically enriched BaCO_3_ can be employed to simplify target preparation to boost ^133^La yields and selectivity from co-produced ^135^La. Another study irradiated [^135^Ba]BaCO_3_ at a 23.3 MeV proton energy, significantly improving ^133^La/^135^La selectivity relative to natural Ba target material, producing 214 MBq ^133^La with 28 MBq ^135^La using [^135^Ba]BaCO_3_, versus 59 MBq ^133^La with 35 MBq ^135^La using [^nat^Ba]BaCO_3_ [41]. Another approach involved irradiating isotopically enriched [^134^Ba]BaCO_3_ at a proton energy of 22 MeV, with subsequent purification yielding up to 1.2–1.8 GBq [^133^La]LaCl_3_ with 0.4% co-produced ^135^La and a radionuclidic purity of >99.5%. The decay of ^133^La into its long-lived daughter ^133^Ba (t_1/2_ = 10.6 y) resulted in 4 kBq ^133^Ba per 100 MBq ^133^La, which was deemed uncritical concerning dose and waste management [42].

**Figure 1:**
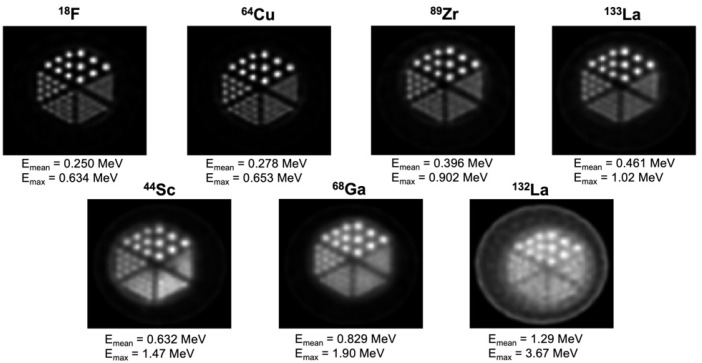
Derenzo phantom PET images reconstructed with MAP for different PET radionuclides, listed in order of increasing positron emission energy. Figure from Nelson et al. [41], with ^18^F, ^64^Cu, ^44^Sc, and ^68^Ga data from Ferguson et al. [46].

As shown in Figure 1, ^133^La PET imaging analysis was performed in Derenzo phantoms and compared with other common PET radionuclides, with ^133^La found to have superior spatial resolution compared to ^44^Sc, ^68^Ga, and another radiolanthanum positron emitter, ^132^La [41].

**Figure 2:**
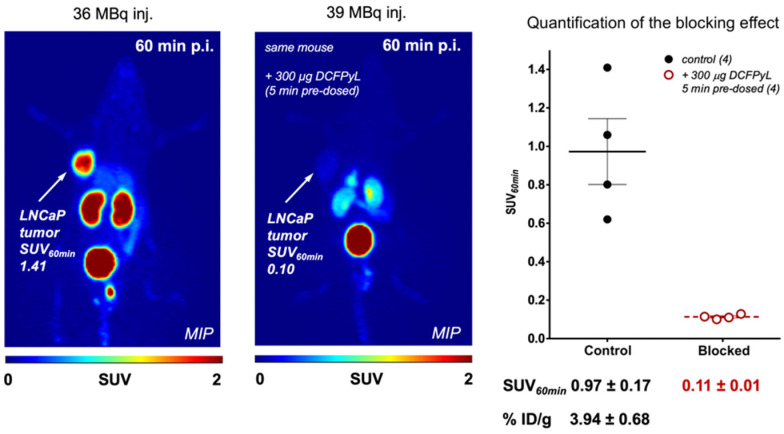
Representative PET images (MIP—maximum intensity projection) at 60 min of [^133^La]La-PSMA-I&T with and without pre-dose of DCFPyL in LNCaP tumor-bearing mice. Figure from Nelson et al. [41].

As depicted in Figure 2, PET imaging was performed with [^133^La]La-PSMA I&T in a prostate cancer mouse model. The LNCaP prostate cancer tumors were delineated with high spatial resolution and minimal off-target uptake, demonstrating the potential for further ^133^La PET imaging applications [41].

Strengths of ^133^La include its 3.9 h half-life that allows sufficient time for separation and distribution to external clinics; a lower positron emission energy compared to ^68^Ga, ^44^Sc, and ^132^La that results in a higher PET imaging spatial resolution [47]; and low energy and intensity co-emitted gamma rays that reduce the radioactive dose. Limitations include the production requirement of medium-energy cyclotron facilities; its lower positron branching ratio of 7.2% that may require additional injected activity relative to other PET radionuclides such as ^18^F; and its decay into relatively long-lived ^133^Ba.

### 3.2. Lanthanum-132 (PET)

Lanthanum-132 (t_1/2_ = 4.6 h) can be produced via the ^132^Ba(p,n)^132^La nuclear reaction using natural Ba metal target material [48,49,50,51]. This beam energy co-produces significant activities of ^135^La and is just below the threshold of the ^133^La production. One study reported yields of 0.26 ± 0.05 MBq·µA^−1^·h^−1 132^La and 5.6 ± 1.1 MBq·µA^−1^·h^−1 135^La for irradiation with 11.9 MeV protons, with ^132^La activity approximately 5% relative to ^135^La activity at the end of bombardment [48,49]. Another study reported yields of 0.8 MBq ^132^La and 17.9 MBq ^135^La for 500 µA·min runs at 11.9 MeV [40]. ^132^La can be purified using DGA resin and complexed with chelators at molar activities suitable for radiopharmaceutical application [49]. A study using a tumor-targeting alkylphosphocholine, NM600, demonstrated significant tumor uptake of [^132^La]La-NM600 and a similar biodistribution to [^225^Ac]Ac-NM600 using PET/CT imaging and ex vivo analysis [48].

Strengths of ^132^La include its 4.6 h half-life, which allows ease of radiopharmaceutical preparation and distribution compared to shorter-lived PET emitters such as ^68^Ga; its stable ^132^Ba decay daughter; and its significant 41.2% positron branching ratio [9]. Limitations include severe cyclotron production constraints owing to the 0.1% natural isotopic abundance of ^132^Ba target material; high energy and intensity co-emitted gamma rays that contribute to excess radioactive dose; and the high maximum positron emission energy of 3.67 MeV, which leads to a low PET spatial resolution and image blurring as shown in Figure 1.

### 3.3. Lanthanum-134/Cerium-134 (PET)

Lanthanum-134 (t_1/2_ = 6.5 min) can be produced via irradiation of natural barium target material; however, its short half-life precludes its direct use for PET imaging. Cerium-134 (t_1/2_ = 3.2 d) decays into ^134^La, permitting an in vivo generator configuration where ^134^Ce can be labelled to a targeting vector, with ^134^La progeny used for PET imaging. Production involves irradiating ^nat^La metal, with yields of 59 MBq·µA^−1^·h^−1^ at proton energies of 62.1–72.1 MeV [52]. A subsequent production route utilized 100 MeV protons to irradiate ^nat^La metal, producing over 3 Ci of ^134^Ce with a 100 µA irradiation for 30 h. Chemical purification can be performed with Bio-Rad AGMP-1 resin, where ^134^Ce is eluted with 0.05 M HNO_3_. ^134^Ce can then be used to label DTPA in its 3+ oxidation state, allowing ^134^Ce to act as a ^225^Ac imaging surrogate, while ^134^Ce can label 3,4,3-LI(1,2-HOPO) in its 4+ oxidation state and act as a ^227^Th imaging surrogate [53,54]. A PET imaging phantom study investigating the spatial resolution and recovery coefficient of ^134^La was found to be inferior and similar to ^18^F, respectively [52].

Strengths of ^134^Ce/^134^La include the 3.2 d half-life of ^134^Ce, which permits PET imaging at extended time points after injection to track ^225^Ac and ^227^Th radiopharmaceuticals; the significant 63.6% positron branching ratio of ^134^La [9]; the stable ^134^Ba decay daughter of ^134^La; and the ability for ^134^Ce to act as a surrogate for both ^225^Ac and ^227^Th. Limitations include a scarcity of production facilities capable of achieving a ~100 MeV proton beam energy; the high positron emission energy of ^134^La, which would result in lower PET spatial resolution; unavoidable co-produced radionuclidic impurities (^139^Ce, t_1/2_ = 137.6); and the potential for in vivo ^134^La daughter redistribution following decay from ^134^Ce that could blur PET imaging [9,39].

### 3.4. Actinium-226 (SPECT)

Actinium-226 (t_1/2_ = 29.4 h) can be produced via high-energy proton spallation of a uranium carbide target or lower-energy proton bombardment of ^226^Ra (t_1/2_ = 1600 y) target material. This involved bombarding a uranium carbide target with 480 MeV protons, with ^226^Ac separated using isotope separation online. This approach yielded 33.8 ± 2.7 MBq ^226^Ac for imaging purposes with high radionuclidic purity [55].

An alternative production route could employ ^226^Ra target material and the ^226^Ra(p,n)^226^Ac nuclear reaction on a lower energy proton cyclotron [9,55,56,57].

**Figure 3:**
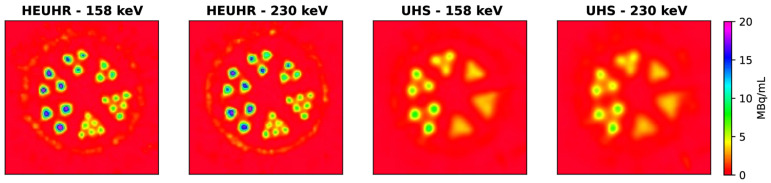
Inter-rod contrast measurements were used to assess image resolution from ^226^Ac SPECT images acquired using two collimators. Figure from Koniar et al. [55].

A phantom assembly with rods between 0.85 and 1.7 mm in diameter and a microSPECT/CT system was used to assess resolution using a high-energy ultra-high resolution (HEUHR) collimator and an extra ultra-high sensitivity (UHS) collimator. The primary 158 keV and 230 keV gamma photopeaks were reconstructed, with the 158 keV photopeak images demonstrating slightly better contrast recovery. For resolution, as depicted in Figure 3, the HEUHR collimator resolved all rods, while the UHS collimator could only resolve rods >1.3 mm and >1.5 mm for the 158 keV and 230 keV photopeaks, respectively [55]. This demonstrated the feasibility of using ^226^Ac as a SPECT imaging surrogate for ^225^Ac.

Advantages of ^226^Ac include its relatively long 29.4 h half-life compared to ^132^La and ^133^La, permitting imaging at extended time points, and its identical chemical properties to ^225^Ac. Limitations include challenges associated with routine irradiation of hazardous ^226^Ra target material, significant β^−^ co-emissions that would increase patient dose, and its decay to β^−^ emitting ^226^Th (t_1/2_ = 30 min), which further decays via multiple alpha and β^−^ emitting progeny before stabilizing at ^206^Pb [9].

## 4. Theranostic Imaging Surrogates Proposed for Lead-212

Lead-212 (t_1/2_ = 10.6 h) has cultivated a significant interest for TAT due to its payload of one alpha and two β^−^ particles in its decay chain and the rapid decay of its progeny to stable ^208^Pb. A recent study using a ^212^Pb somatostatin analogue demonstrated a significant antitumor effect in patients with metastatic neuroendocrine tumors, and additional radiopharmaceuticals are under development to treat other cancers [1,58,59,60,61,62]. Production of ^212^Pb involves synthesizing its parent radionuclide, ^228^Th (t_1/2_ = 1.9 y), via ^226^Ra irradiation in a nuclear reactor or high-energy proton spallation of ^232^Th target material. ^212^Pb can then be extracted in a convenient generator setup from ^228^Th or one of its intermediate progeny, ^224^Ra (t_1/2_ = 3.6 d) [12,63,64,65,66,67].

Previous clinical trials have employed imaging techniques with conventional radiometals such as ^68^Ga [58]. While direct SPECT imaging of ^212^Pb can be performed using its 239 keV (44%) gamma emissions [9], it is desirable to have an imaging surrogate that can be used for research owing to the limited supply of ^212^Pb and to provide the most accurate pre-therapy scans to assess patient eligibility for ^212^Pb TAT radiopharmaceuticals. While no positron-emitting Pb isotopes are suitable for use as ^212^Pb imaging surrogates, multiple gamma-ray emitters exist, with ^203^Pb being a prime candidate for SPECT imaging.

### Lead-203 (SPECT)

Lead-203 (t_1/2_ = 51.9 h) emits X-rays and a primary 279 keV (81%) gamma photon that can be used for SPECT imaging. ^203^Pb has been synthesized via ^203^Tl(p,n)^203^Pb, ^203^Tl(d,2n)^203^Pb, and ^205^Tl(p,3n)^203^Pb nuclear reactions on cyclotrons [21,45,63,64,68,69,70,71]. Natural thallium metal can be used as a target material; however, significant precautions must be taken owing to the high toxicity of Tl, and its low thermal conductivity and melting point (304 °C) that makes it prone to melt or sublime under intense heat of a cyclotron beam. Natural Tl metal has been used as a target material, with one technique bombarding ^nat^Tl at 25–26 MeV, producing up to 21 GBq ^203^Pb five days after end of bombardment [61]. However, irradiating ^nat^Tl produces significant activities of ^201^Pb (t_1/2_ = 9.3 h), which must be permitted to decay significantly to achieve a ^203^Pb product with high radionuclidic impurity. ^203^Pb can be produced at lower proton energies using natural or isotopically enriched ^203^Tl and the ^203^Tl(p,n)^203^Pb nuclear reaction ^63,71^, with one process yielding up to 138.7 ± 5.1 MBq ^203^Pb [64]. However, yields are limited due to the low nuclear reaction cross-section in this energy window [45]. Alternatively, isotopically enriched ^205^Tl can be irradiated at 23–24 MeV proton energies to produce ^203^Pb via the ^205^Tl(p,3n)^203^Pb reaction. This produces significant activities of ^203^Pb (>12 GBq at the end of purification) with a high radionuclidic purity (>99.9%) made possible by the near absence of ^203^Tl and its resulting ^201^Pb co-production ^21,63^. Enriched ^203^Tl can also be bombarded with deuterons to produce ^203^Pb via the ^203^Tl(d,2n)^203^Pb reaction; however, this production route has a lower maximum cross-section compared to the ^205^Tl(p,3n)^203^Pb reaction, and ^203^Tl (29.5% natural isotopic abundance) is more expensive to enrich than ^205^Tl (70.5% natural isotopic abundance).

^203^Pb can be separated using ion exchange resins such as Pb resin, carboxymethyl resin, and Dowex-1X8 anion exchange resin. This can yield a concentrated ^203^Pb product in [^203^Pb]PbCl_2_ or [^203^Pb]Pb(OAc)_2_, with direct and rapid room temperature radiolabeling of [^203^Pb]Pb(OAc)_2_ using chelators such as DOTA, PSC, and TCMC. Radiolabeling achieves very high molar activities, and ^203^Pb chelate complexes have been shown to be highly stable in human serum up to 120 h [21,63,64,69,70].

**Figure 4:**
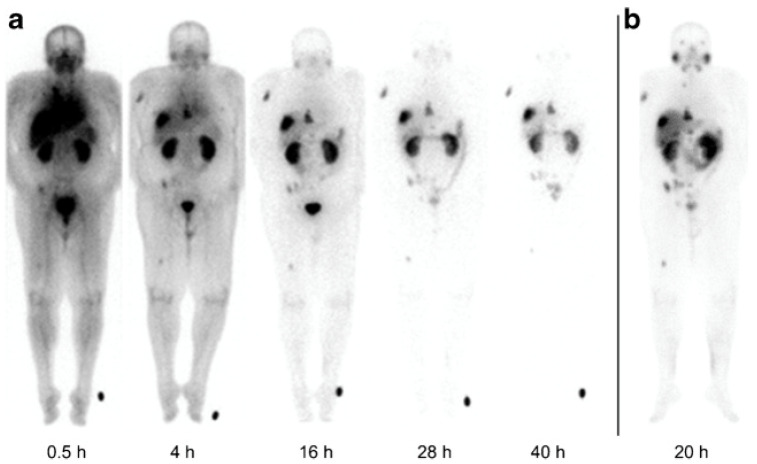
Planar scans of a PSMA targeting ligand [^203^Pb]Pb-CA012 (**a**), versus a [^177^Lu]Lu-PSMA 617 treatment scan (**b**). Figure from dos Santos et al. [74].

Phantom imaging of ^203^Pb has been performed, with imaging spatial-resolution results comparable to ^99m^Tc for 1.6–4.8 mm diameter fillable rod regions [72]. In vivo preclinical and clinical SPECT imaging of uncomplexed and chelated ^203^Pb has been performed [71,73]. Studies have included ^203/212^Pb-labeled PSMA and gastrin-releasing peptide receptor-targeting agents for imaging and radiotherapy of prostate-cancer-bearing mice [60,61,74,75], and ^203/212^Pb-labeled anti-melanin antibodies and melanocortin subtype 1 receptor targeting ligands for imaging and therapy of melanoma-bearing mice [59,72,73,76,77,78,79]. As shown in Figure 4, a PSMA-targeting ^203^Pb agent, [^203^Pb]Pb-CA012, exhibited a comparable biodistribution to [^177^Lu]Lu-PSMA 617 with high tumor uptake relative to other tissues [74].

Strengths of ^203^Pb include its relatively long 51.9 h half-life, which permits imaging at extended time points to inform ^212^Pb TAT dosimetry; its relatively clean X-ray and gamma photon emission spectrum that enables SPECT imaging using a low or high-energy collimator; its ability to rapidly and stably radiolabel targeting vectors under mild chemical conditions at room temperature (similar to ^212^Pb); and established production processes that provide ^203^Pb with high radionuclidic purity in yields suitable for multiple patients per production run. Limitations include risks associated with preparing and irradiating highly toxic thallium targets and potential uncertainties with using ^203^Pb pharmacokinetic data for ^212^Pb therapy planning due to the release of ^212^Bi progeny during ^212^Pb decay [80].

## 5. Theranostic Imaging Surrogates Proposed for Radium-223/224

Radium-223 (t_1/2_ = 11.4 d) is used as an alpha therapy for men with bone-metastatic castration-resistant prostate cancer. It works as a calcium-mimetic by accumulating in and irradiating osteoblastic lesions, while sparing most surrounding healthy tissue [81]. It is the only FDA-proved alpha-particle-emitting radiopharmaceutical (Xofigo^®^) and has been used to treat over 18,000 patients since 2013 [82]. However, unlike targeted alpha therapy, ^223^Ra is currently administered as a [^223^Ra]RaCl_2_ salt in an aqueous buffer without a chelator or biological-targeting agent. Therefore, the established clinical efficacy and safety of ^223^Ra makes it an attractive TAT candidate [82]. Similarly, ^224^Ra (t_1/2_ = 3.6 d) has been employed in a dual targeting strategy with ^212^Pb, where ^224^Ra accumulates at primary bone cancer sites or bone metastases, while extra-skeletal metastases can be targeted with a ^212^Pb-labeled cancer-specific vector [83,84]. [^224^Ra]RaCl_2_ (marketed as ^224^SpondylAT^®^ (Eckert & Ziegler, Berlin, Germany) has also been used to treat bone and joint disease, ankylosing spondylitis [85], while ^224^Ra is also under investigation for a novel brachytherapy called diffusing alpha-emitter radiation therapy (DaRT). In DaRT, ^224^Ra-infused seeds are inserted into solid tumors, which are then irradiated with alpha emissions released during the diffusion and subsequent decay cascade of its ^220^Rn progeny [86,87,88,89,90,91,92,93,94,95]. Both ^223^Ra and ^224^Ra are currently produced in significant activities as by-products and decay daughters of neutron irradiation of ^226^Ra in a nuclear reactor. With proven purification techniques, this positions these radionuclides well for TAT [67,96,97].

^223^Ra has recently been stably complexed with the chelator macropa, where a [^223^Ra]Ra–macropa complex exhibited rapid clearance and low ^223^Ra bone absorption, suggesting in vivo stability. This has opened the possibility of using ^223^Ra complexed using functionalized chelators to target metastases beyond the bone, similar to other radionuclides used in targeted alpha therapy [82,98].

While ^223^Ra possesses several gamma emissions within an energy window suitable for SPECT imaging (^223^Ra: 269 keV, (13%); 154 keV (6%); ^224^Ra: 241 keV (4.1%)), the low intensity of these gamma photons would likely be insufficient to generate a high-quality SPECT image when considering the relatively low injected therapeutic activity (~50 kBq/kg) injected [9,81]. Similarly, a relatively low injection activity of ^224^Ra due to its 3.6 d half-life could complicate direct SPECT imaging. Therefore, an imaging surrogate is desirable to assess the viability of ^223/224^Ra radiopharmaceuticals, with ^131^Ba emerging as a candidate.

### Barium-131 (SPECT)

Barium-131 (t_1/2_ = 11.5 d) decays via electron capture to ^131^Cs (t_1/2_ = 9.7 d) and subsequently to stable ^131^Xe, emitting gamma rays suitable for SPECT imaging (496 keV (48%); 216 keV (20%); 124 keV (30%); 371 keV (14%)) [9]. Additionally, approaches designed to sequester Ra (nanoparticles, chelation via macropa or ligands based on the arene scaffold) [99,100] should be transferrable owing to the proven use of Ba as a non-radioactive surrogate for Ra [101]. Therefore, the favorable imaging emissions of ^131^Ba compared to other Ba radionuclides (^135m^Ba, ^133m^Ba), and the similar half-life and chemistry of ^131^Ba to ^223/224^Ra positions ^131^Ba as a promising surrogate to track in vivo ^223/224^Ra biodistribution.

^131^Ba can be produced via neutron irradiation of isotopically enriched ^130^Ba (natural abundance = 0.1%) in a nuclear reactor, which would co-produce significant activities of ^133^Ba [45,102]. Alternatively, ^131^Ba can be produced via proton irradiation of natural cesium target material in a cyclotron via the ^133^Cs(p,3n)^133^Ba nuclear reaction with a small ^133^Ba contamination (0.01%) at beam energies of 27.5 MeV [45,101]. A 4 h irradiation yielded 190 ± 26 MBq ^131^Ba, and an SR resin was used to separate ^131^Ba from the Cs target material. ^131^Ba was subsequently successfully radiolabeled to macropa, and exhibited stability in human serum [101].

**Figure 5:**
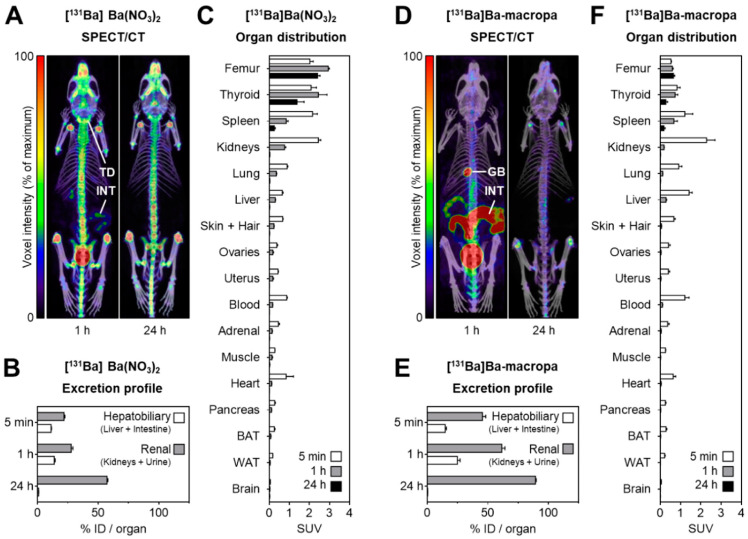
(**A**) SPECT/CT images of [^131^Ba]Ba(NO_3_)_2_; (**B**,**C**) excretion profile and organ distribution of [^131^Ba]Ba(NO_3_)_2_; (**D**) SPECT/CT images of [^131^Ba]Ba-macropa; and (**E**,**F**) excretion profile and organ distribution of [^131^Ba]Ba-macropa [101].

SPECT imaging was performed in a cylindrical syringe, which enabled visualization of the radionuclide distribution. However, image quality was limited due to artifacts caused by the higher energy gamma photon emissions. As highlighted in Figure 5, small animal SPECT/CT was performed with [^131^Ba]Ba(NO_3_)_2_, showing ^131^Ba accumulation within the entire skeleton 1 h post-injection, which was still present 24 h after injection. Additional SPECT imaging was performed with [^131^Ba]Ba-macropa, with rapid clearance observed through the intestines and gallbladder [101]. This demonstrated the feasibility of using ^131^Ba as a SPECT imaging surrogate for ^223/224^Ra.

Advantages of ^131^Ba include its relatively long half-life, which is similar to ^223^Ra, permitting imaging at extended time points; the ability to sequester ^131^Ba in the macropa chelator similar to ^223^Ra; and established ^131^Ba production routes. Limitations include higher energy gamma photon emissions, which increase unintended patient dose and can cause image artifacts. The presence of co-produced ^133^Ba may also require additional dosimetric analysis. Additionally, the decay of ^131^Ba to ^131^Cs with X-ray emissions adds a suboptimal patient radioactive dose compared to an imaging radionuclide with direct decay to stable progeny. Finally, further improvements in the cyclotron production route would be required to synthesize enough activity for multiple patients in a single batch.

## 6. Theranostic Imaging Surrogates Proposed for Astatine-211

Astatine-211 (t_1/2_ = 7.2 h) has garnered interest for TAT owing to its decay to either ^207^Bi (t_1/2_ = 31.6 y) via alpha emission or to ^211^Pb via electron capture followed by alpha decay to stable ^207^Pb [9]. Therefore, each ^211^At decay yields one alpha particle. The ^211^At decay chain also emits few high-energy gamma photons, which avoids excess radiation dose [8]. ^211^At can be produced in medium-energy alpha cyclotrons using bismuth target material and the ^209^Bi(α,2n)^211^At nuclear reaction or via heavy ion irradiation and the ^209^Bi(^7^Li,5n)^211^Rn reaction, where ^211^At is obtained via decay of its longer-lived parent ^211^Rn (t_1/2_ = 14.6 h) in a generator configuration [8,103,104]. Production yields of up to 6.6 GBq have been reported, which would be sufficient for clinical radiopharmaceutical production for several patients and distribution several hours from the production site [8,105].

^211^At was initially investigated for treating thyroid disorders and is currently being evaluated in clinical trials for multiple myeloma, leukemia, myelodysplastic syndromes, thyroid cancer, and malignant pheochromocytoma [106]. While direct SPECT imaging of ^211^At is possible using the X-rays emitted during ^211^At decay to ^211^Po, it is desirable to have an imaging surrogate to perform pre-therapy assessment scans and research, owing to the limited supply and short half-life of ^211^At that generally precludes its use at facilities located more than several hours from a production site. Several candidates exist for use as ^211^At diagnostic imaging surrogates: chemically identical ^209^At, or chemically similar ^123^I, ^124^I and ^131^I.

### 6.1. Iodine-123 (SPECT)

Iodine-123 (t_1/2_ = 13.2 h) decays via electron capture to near-stable ^123^Te, and is commonly used in nuclear medicine and research of various malignancies and biological processes, including thyroid diseases and tumor imaging [107]. Its X-ray emissions and primary gamma photopeak of 159 keV (83.6%) are well suited for SPECT imaging [9].

^123^I is primarily produced via the ^124^Xe(p,2n)^123^I nuclear reaction using a highly enriched ^124^Xe gas target, which enables ^123^I production with a high yield and radionuclidic purity. The subsequent ^123^I product is commercially available in dilute NaOH solutions [108,109].

Strengths of ^123^I include its favorable emission spectrum for SPECT imaging, similar half-life relative to ^211^At, and commercial availability. Limitations include hazards associated with volatile radioactive products, the lower image quality of SPECT images to PET imaging, and the low natural abundance (0.095%) of ^124^Xe target material.

### 6.2. Iodine-124 (PET)

Iodine-124 (t_1/2_ = 4.2 d) undergoes positron decay to stable ^124^Te and is employed for PET imaging studies. Its relatively long half-life allows extended radiosynthesis, quantitative imaging over several days, and distribution to sites far from production facilities [9]. ^124^I is typically produced using isotopically enriched ^124^Te and the ^124^Te(d,2n)^124^I or ^124^Te(p,n)^124^I nuclear reactions [110,111]. Applications in nuclear medicine and research have been extensive, including thyroid and parathyroid imaging, studies of neurotransmitter receptors, and monoclonal antibody imaging in cancer [110].

Strengths of ^124^I include its long half-life that eases logistics and allows imaging at extended time points. Limitations include hazards associated with volatile radioactive products; a relatively low positron branching ratio (22.7%); relatively high average positron emission energy (E_mean_ = 820 keV) that results in a lower spatial resolution compared to other PET radionuclides; and co-emitted gamma rays (603 keV (63%), 1691 keV (11%)) that increase dose and shielding requirements [9].

### 6.3. Iodine-131 (SPECT)

Iodine-131 (t_1/2_ = 8.0 d) undergoes β^−^ decay to stable ^131^Xe, and similar to ^123^I and ^124^I, it is primarily used for treating thyroid malignancies [107]. ^131^I can be produced in a nuclear reactor by irradiating either ^130^Te or uranium targets [112].

Strengths of ^131^I include its 8 d half-life that permits imaging at extended time points, commercial availability, and primary 364 keV (81.5%) gamma emission that is well suited for SPECT imaging. However, limitations include hazards associated with volatile radioactive products and significant β^−^ emissions that would increase patient dose [9].

### 6.4. Astatine-209 (SPECT)

**Figure 6:**
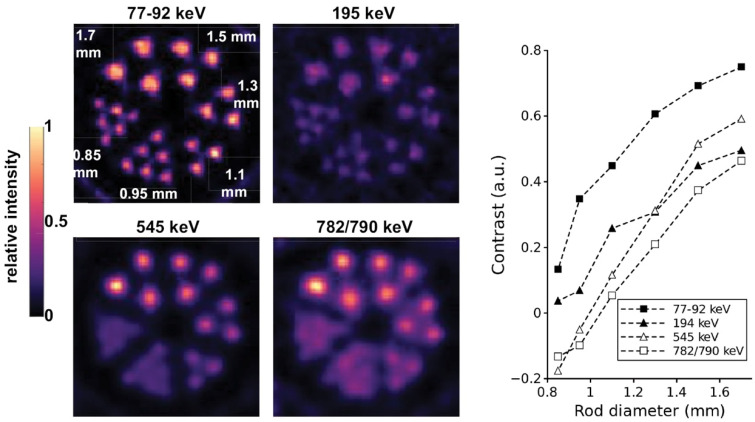
SPECT images and inter-rod contrast data for a phantom containing ^209^At [114].

Astatine-209 (t_1/2_ = 5.4 h) decays via alpha emissions (4%) to ^205^Bi (t_1/2_ = 14.9 d) followed by decay to stable ^205^Pb, or via electron capture (96%) to ^209^Po (t_1/2_ = 124 y). During decay to ^209^Po, X-rays and gamma emissions (545 keV (91.0%), 195 keV (22.6%), and 239 keV (12.4%) enable SPECT imaging. ^209^At can be produced via high-energy proton spallation of a uranium carbide target, followed by online surface ionization and A = 213 isobars separation. This can yield ^209^At in activities on the order of 10^2^ MBq [113]. Subsequent chemical purification employs a Te column to obtain purified ^209^At [113,114]. As shown in Figure 6, subsequent studies using ^209^At for phantom imaging demonstrated that image reconstruction with ^209^At X-ray emissions was superior to using its gamma emissions [114]. Additionally, in vivo imaging measurements of ^209^At uptake in mice matched ex vivo measurements within 10%. This demonstrated the potential of using ^209^At to accurately determine astatine biodistributions [114].

Strengths include identical chemistry to ^211^At, which would give more certainty to ^209^At pharmacokinetic data. Limitations include alpha emissions in ^209^At decay that would require dosimetric evaluation; numerous high-energy gamma rays that complicate shielding and increase patient dose; the need to consider longer-lived ^205^Bi in dosimetry evaluations; and production/logistical challenges associated with distributing relatively short-lived ^209^At from a limited number of facilities capable of high-energy proton spallation and separation of ^211^At from actinide targets [8].

## 7. Theranostic Imaging Surrogates Proposed for Thorium-227

Thorium-227 (t_1/2_ = 18.7 d) decays via alpha emission to ^223^Ra and can be harvested from a generator containing ^227^Ac (t_1/2_ = 21.8 y) that is produced via nuclear reactor irradiation of ^226^Ra [115]. Thorium can be complexed with octadentate 3,2-hydroxypyridinone (3,2-HOPO) chelators attached to biological-targeting vectors ^115^. Ongoing clinical studies involving ^227^Th TAT include targeting tumors expressing human epidermal growth factor receptor 2 (HER2), PSMA, mesothelin (MSLN), and CD22 [116]. ^227^Th does emit a 236 keV (12.9%) gamma photon that would be suitable for SPECT imaging. However, the long half-life of ^227^Th relative to other TAT radionuclides would likely result in a low injected therapeutic activity, which could be insufficient for direct imaging ^9^. Therefore, an imaging surrogate to assess ^227^Th radiopharmaceutical pharmacokinetics is desirable, with the ^134^Ce/^134^La PET imaging pair showing promise (see Section 3.3). A significant uncertainty of using any theranostic imaging pair with ^227^Th involves its long-lived ^223^Ra progeny, which has the potential for substantial redistribution and alpha irradiation of healthy tissue after decay from ^227^Th. This would significantly complicate direct comparisons between imaging and inferred therapeutic dosimetry and require further study.

## 8. Theranostic Imaging Surrogates Proposed for Terbium-149

Terbium-149 (t_1/2_ = 4.1 h) is a unique radionuclide for TAT. It emits low-energy alpha particles with a short tissue range and decays via several daughter radionuclides to stable ^145^Nd and ^141^Pr, without any subsequent alpha emissions [9]. This absence of alpha-emitting progeny is regarded as a potential strength for ^149^Tb TAT. ^149^Tb is produced via high-energy proton spallation of a tantalum target followed by online isotope separation or ^3^He bombardment of a ^151^Eu target [19,20,117,118]. 100 MBq of ^149^Tb was obtained in a solution suitable for preclinical applications and successfully labeled to a DOTANOC targeting vector [118]. While PET images were successfully obtained using [^149^Tb]Tb-DOTANOC in a mouse model, ^149^Tb possesses a relatively low positron branching ratio (21%) and relatively high positron emission energy (E_mean_ = 805 keV). These physical factors could present challenges to obtaining high-quality clinical PET images. Additionally, due to limited production and the resulting extreme scarcity of ^149^Tb, imaging surrogates would be helpful research tools to evaluate its potential for TAT. Two surrogate candidates are ^155^Tb and ^152^Tb.

### 8.1. Terbium-155 (SPECT)

Terbium-155 (t_1/2_ = 5.3 d) decays via electron capture to stable ^155^Gd, with X-ray and gamma-ray emissions including 87 keV (32%), 105 keV (25%), 180 keV (7.5%), and 262 keV (5%) [9]. ^155^Tb can be produced via the ^156^Gd(p,2n)^155^Tb reaction at 23 MeV, or the ^155^Gd(p,n)^155^Tb reaction at 10 MeV [119]. The ^156^Gd(p,2n)^155^Tb has higher demonstrated production yields (up to 1.7 GBq); however, it has a lower radionuclidic purity compared to the final product of the ^155^Gd(p,n)^155^Tb reaction (200 MBq yield). Subsequently, phantom and in vivo SPECT/CT studies were successfully performed with [^155^Tb]Tb-DOTATOC, demonstrating a similar image quality to ^111^In [119,120].

Advantages of ^155^Tb include its accessible production routes that can synthesize multi-patient activities per run, decay to stable ^155^Gd, and its long half-life that enables long-duration imaging. Limitations include relatively low imaging performance compared to other diagnostic radionuclides, such as PET emitters.

### 8.2. Terbium-152 (PET)

Terbium-152 (t_1/2_ = 17.5 h) decays via positron emission to near-stable ^152^Gd with a positron branching ratio of 20.3% and an average positron energy of 1140 keV [121]. Several primary co-emitted gamma rays include 344 keV (63.5%), 271 keV (9.5%), 586 keV (9.2%), and 779 keV (5.5%). ^152^Tb synthesis is extremely limited, with the existing production route involving high-energy proton spallation of a tantalum target at 1.4 GeV and online isotope separation [122]. Following chemical separation, phantom studies revealed increased image noise due to the smaller positron branching ratio of ^152^Tb, and subsequently [^152^Tb]Tb-DOTANOC was administered to a patient and used to acquire PET scans [121].

Advantages of ^152^Tb include a relatively long half-life permitting imaging at extended time points and its decay to near-stable ^152^Gd. Limitations include the scarcity of facilities capable of achieving proton energies for production, the higher average positron emission energy, and significant co-emitted gamma rays that increase the radioactive dose.

## 9. Summary and Outlook for Alpha-Emitter Imaging Surrogates

As highlighted in this article, multiple SPECT and PET imaging surrogates have demonstrated the potential to enhance clinical TAT applications and research. Table 1 presents a summary of proposed theranostic imaging surrogates for alpha emitters, along with their properties and production status.

Production capabilities must be augmented to enable more patients and research efforts to benefit from TAT imaging surrogates. Existing medium-energy cyclotron facilities are well positioned to improve the supply chain of imaging surrogates such as ^133^La, ^203^Pb, and ^155^Tb by adapting and optimizing established production techniques to the unique capabilities of each facility. A stable supply of isotopically enriched accelerator target material will be required to support growing production efforts for many of these radionuclides. Other imaging surrogates such as ^226^Ra, ^152^Tb, ^209^At, and ^134^Ce/^134^La require high-energy accelerators, bombarding hazardous target material, and techniques such as mass separation to enable their production. While these surrogates have demonstrated research potential, their widespread deployment for radiopharmaceutical development and clinical application may be limited owing to the scarcity of facilities capable of their production.

Except for ^149^Tb, which possesses a single alpha emission in its decay chain, most TAT radionuclides, including ^225^Ac, ^212^Pb, ^223^Ra, ^224^Ra, ^227^Th, and ^211^At, possess a cascade of decay progeny that are released from the original target site due to recoil energy and deposit additional alpha radiation in surrounding healthy tissues. While the highlighted imaging surrogates are well positioned to provide more accurate dosimetry data for the TAT parent radionuclide decay, there will be a degree of uncertainty regarding the dose from alpha-emitting decay progeny. This uncertainty will depend on the type of malignancy, internalization within targeted cells, and other factors within the disease microenvironment that influence the radiopharmaceutical pharmacokinetics. However, this limitation does not negate the improved accuracy of biodistribution dosimetry data conferred by using imaging surrogates matched to the TAT parent radionuclide, particularly when radionuclides are stably bound to their targeting vector. Therefore, TAT imaging surrogates have the potential to assist the preclinical development and clinical deployment of TAT radiopharmaceuticals and represent a significant improvement over conventional PET and SPECT imaging radionuclides currently paired with TAT.

## 10. Conclusions

Recent preclinical and clinical advances in targeted alpha therapy have spurred significant interest in utilizing alpha-emitting radiopharmaceuticals to treat metastatic cancers and other malignancies. Despite their strong potential, TAT radiopharmaceuticals suffer from an acute supply shortage of alpha-emitting radionuclides due to production constraints. This severely restricts the availability for patient therapy and slows the development of new TAT radiopharmaceuticals. Additionally, many alpha-emitting radionuclides do not possess radioactive emissions suitable for diagnostic imaging. This often leads to diagnostic radiopharmaceuticals being employed with suboptimally paired imaging radionuclides that possess different chemistries from their therapeutic counterpart, which can potentially result in different radiopharmaceutical biodistributions. Therefore, increasing the availability of SPECT and PET imaging TAT surrogates has strong potential to improve the accuracy of dosimetry and treatment tracking, and enhance TAT research output by using more economical and less potent diagnostic radionuclides for preclinical radiopharmaceutical development. Therefore, TAT imaging surrogates hold potential to improve the accuracy of diagnostic scans, equipping clinicians and researchers with more accurate biodistribution and dosimetry data that they can use to expedite the development and deployment of novel TAT radiopharmaceuticals.

## Figures and Tables

**Table 1:** Summary of prominent TAT radionuclides and their proposed theranostic SPECT and PET imaging surrogates.

Alpha Emitter	Proposed Imaging Surrogate	Half-Life	Key Decay Progeny	Key Imaging Emissions	Primary Production Routes	Production Status and References
^225^Ac		9.9 d	^211^Fr, ^217^At, ^213^Bi, ^213^Po, ^209^Tl, ^209^Pb, ^209^Bi (stable)	γ: 100 keV (1%), 218 keV (11.4%)	^229^Th generator, ^226^Ra proton/photonuclear reactions, ^232^Th spallation	Routine production [31,32,33,34]
	^133^La	3.9 h	^133^Ba	β^+^: 460 keV (mean), 7.2%	^135^Ba or ^134^Ba proton irradiation	Research [40,41,42]
	^132^La	4.8 h	^132^Ba (stable)	β^+^: 1290 keV (mean), 42.1%	^132^Ba proton irradiation	Research [48,49,50]
	^134^Ce/^134^La	3.2 d/6.5 min	^134^Ba (stable)	β^+^: 1217 keV (mean), 63.6%	High-energy ^139^La proton irradiation	Research [52,53,54]
	^226^Ac	29.4 h	^226^Ra, ^226^Th, ^222^Ra, ^218^Rn, ^214^Po, ^210^Pb, ^210^Bi, ^210^Po, ^206^Pb (stable)	γ: 230 keV (26.9%), 158 keV (17.5%)	^226^Ra proton irradiation	Research [55]
^212^Pb		10.6 h	^212^Bi, ^212^Po, ^208^Tl, ^208^Pb (stable)	γ: 239 keV (44%)	^228^Th generator	Routine production [12,63,64,65,66,67]
	^203^Pb	51.9 h	^203^Tl (stable)	γ: 279 keV (81%) X-ray: 73 keV (37%), 71 keV (22%)	^205^Tl proton irradiation, ^203^Tl proton or deutron irradiation	Routine production [21,63,64,68,69,70,71]
^223^Ra		11.4 d	^219^Rn, ^215^Po, ^215^At, ^211^Pb, ^211^Bi, ^211^Po, ^207^Tl, ^207^Pb (stable)	γ: 269 keV (13%), 154 keV (6%)	^226^Ra nuclear reactor irradiation	Routine production [67,96,97]
^224^Ra		3.6 d	^220^Rn, ^216^Po, ^212^Pb, ^212^Bi, ^212^Po, ^208^Tl, ^208^Pb (stable)	γ: 241 keV (4%)	^228^Th generator	Routine production [67,96,97]
	^131^Ba	11.5 d	^131^Cs	γ: 496 keV (48%), 124 keV (30%), 216 keV (20%), 371 keV (14%)	^133^Cs proton irradiation	Research [101,102]
^211^At		7.2 h	^207^Bi, ^211^Po, ^207^Pb (stable)	X-ray: 79 keV (21%)	^209^Bi alpha particle irradiation	Routine production [8,103,104,105]
	^123^I	13.2 h	^123^Te (near stable)	γ: 159 keV (83.6%)	^124^Xe proton irradiation	Routine production [108,109]
	^124^I	4.2 d	^123^Te (stable)	β^+^: 820 keV (mean), 22.7%	^124^Te proton or deutron irradiation	Routine production [110,111]
	^131^I	8.0 d	^131^Xe (stable)	γ: 364 keV (89.6%)	^130^Te or uranium nuclear reactor irradiation	Routine production [112]
	^209^At	5.4 h	^209^Po, ^209^Bi, ^205^Bi, ^205^Pb, ^205^Tl	γ: 545 keV (91%), 239 keV (12.4%), 195 keV (22.6%)	Proton spallation of uranium carbide	Research [113,114]
^227^Th		18.7 d	^223^Ra, ^219^Rn, ^215^Po, ^215^At, ^211^Pb, ^211^Bi, ^211^Po, ^207^Tl, ^207^Pb (stable)	γ: 235 keV (12.9%)	^226^Ra nuclear reactor irradiation	Routine production [115]
	^134^Ce/^134^La	3.2 d/6.5 min	^134^Ba (stable)	β^+^: 1217 keV (mean), 63.6%	High-energy ^139^La proton irradiation	Research [52,53,54]
^149^Tb		4.1 h	^149^Gd, ^149^Eu, ^149^Sm (stable), ^145^Eu, ^145^Sm, ^145^Pm, ^145^Nd (stable)	β^+^: 720 keV (mean), 7.1% γ: 165 keV (26.4%)	^151^Eu helium-3 bombardment, proton spallation of Ta	Research [19,20,117,118]
	^155^Tb	5.3 d	^155^Gd (stable)	γ: 87 keV (32%), 105 keV (25%), 180 keV (7.5%), and 262 keV (5%).	^155^Gd proton irradiation	Research [119]
	^152^Tb	17.5 h	^152^Gd (near stable)	β^+^: 1140 keV (mean), 20.3%	Proton spallation of Ta	Research [122]

## Data Availability

Data sharing is not applicable.
